# Assessing Family Functioning Before and After an Integrated Multidisciplinary Family Treatment for Adolescents With Restrictive Eating Disorders

**DOI:** 10.3389/fpsyt.2021.653047

**Published:** 2021-06-04

**Authors:** Martina M. Mensi, Marika Orlandi, Chiara Rogantini, Livio Provenzi, Matteo Chiappedi, Michela Criscuolo, Maria C. Castiglioni, Valeria Zanna, Renato Borgatti

**Affiliations:** ^1^Child Neurology and Psychiatry Unit, IRCCS Mondino Foundation, Pavia, Italy; ^2^Department of Brain and Behavioural Sciences, University of Pavia, Pavia, Italy; ^3^Anorexia Nervosa and Eating Disorder Unit, Bambino Gesù Children Hospital IRCCS, Rome, Italy

**Keywords:** adolescence, eating disorders, family functioning, Lausanne Trilogue Play, family therapy, multi-professional treatment

## Abstract

The present study presents an investigation of family functioning in the families of adolescents with severe restrictive eating disorders (REDs) assessed before and 6 months after a multidisciplinary family treatment program that combined psychodynamic psychotherapy, parental role intervention, and triadic or family-centered interventions. Nutritional counseling and neuropsychiatric monitoring of the overall treatment and care process were also provided. Family functioning was assessed using the clinical version of the Lausanne Trilogue Play (LTPc), a semi-structured procedure for observing family dynamics, previously validated for this patient population. The LTPc is divided into four phases. In phase 1, the mother interacts with the patient while the father assumes the role of observer. In phase 2, the father plans an activity with the patient while the mother observes. In phase 3, all the family members interact. Finally, in phase 4, the parents talk while the adolescent observes. A significant change emerged in family functioning after the treatment, but only for the interactive phase 2, when the father is required to interact with the daughter while the mother silently observes. The results of this study suggest that a relatively brief multidisciplinary treatment program may significantly improve family functioning in the families of patients diagnosed with severe REDs. Although appropriate clinical trials are needed to further test the efficacy of this treatment, the results also reinforce the concept that treatment programs targeting the individual patient and both the parents should be a first-line approach in adolescents with severe REDs.

## Introduction

Restrictive eating disorders (REDs) are a heterogeneous group of potentially severe psychopathological conditions that have shown an increased incidence among young people in recent years, especially in the high-risk group of 15- to 19-year-old girls (1–3). REDs are thought to have a multifactorial etiology involving individual vulnerability factors influenced by biological, psychological, environmental, and family-related factors (4–8).

Among the latter, previous research has highlighted that family relations are frequently dysfunctional in the families of individuals affected by REDs (9–11). Cerniglia and his group (12), for example, underlined difficulties in respecting interpersonal boundaries, poor tolerance of conflict, and low satisfaction. Use of the clinical version of the Lausanne Trilogue Play (LTPc) (13)—a semi-structured method for observing family dynamics—may help to identify specific characteristics of a family's triadic interactions that may be linked to the patient's clinical condition. Previous LTPc studies have in fact highlighted dysfunctional interaction patterns in the families of individuals with REDs (14–16). For example, fathers were found to experience specific difficulties in maintaining a scaffolding role in relation to their daughters' development, and in providing them with support and guidance (14, 15, 17). This is line with current literature (18, 19) showing that fathers tend to disengage from caregiving. Accordingly, it has been suggested that greater affective engagement and participation in the healthcare process on the part of fathers should be encouraged (20, 21). During the last decade, the focus of family functioning research in this specific area has shifted away from the role of family-related factors in maintaining REDs to enhancement of protective family factors that may improve interventions (22). In these families, parents often tend to adapt their own lives to the RED symptoms of their daughters; for example, they may accept meal rituals in order to avoid conflicts (23). Not surprisingly, therefore engagement of the whole family in the adolescent's treatment and care process is now recognized as a key prognostic factor (4, 7, 17, 24–29).

Family-centered approaches [e.g., family-based therapy (FBT)] (30, 31) are among the most effective (type I evidence) interventions for the psychiatric care of patients with REDs (20, 32); in particular, they are considered the first-line treatment for severe cases in adolescence (25, 33, 34). Recently, FBT has also been found to be effective in the treatment of avoidant/restrictive food intake disorder (ARFID) (35). Nonetheless, the efficacy may be partial when family members are not properly engaged in the treatment and care process (26, 27), and a significant number of patients may not respond well to FBT. Another family therapy approach that has shown good evidence of effectiveness is the psychodynamic model based specifically on intrafamily relationships developed by the French group at the Montsouris Institute in Paris (18). This model focuses more on psychological issues than on eating behavior symptoms. It has been shown to be effective in reducing feeding symptoms and improving general psychopathological functioning, as measured by the Morgan–Russell Outcome Assessment Schedule (MROAS) (36) adapted for adolescent patients (37). These results suggest that improving family functioning may be an intermediate goal, important in promoting better clinical outcomes in the adolescent herself (17, 38, 39). Individual approaches, such as adolescent-focused therapy (40), can also be effective when patients are affected by more severe psychopathological conditions and when their autonomy is severely compromised. Reinstating adaptive psychological development trajectories should be considered a pivotal aim to target within the recovery process (39, 41). However, when family relationships are highly dysfunctional, individual psychotherapy can achieve only partial results; dysfunctional parenting may negatively impact the treatment and care process of adolescents with REDs, and may represent a significant obstacle to the effectiveness of individual psychotherapy (18, 41).

On the basis of these premises, and with a view to identifying a suitable treatment for patients with severe REDs, a multidisciplinary family therapy approach integrating the models by Godart et al. (18) and Fitzpatrick et al. (40) was developed at two university tertiary care services in Italy. The treatment program we developed combines principles from various models of intervention (i.e., psychodynamic psychotherapy, parental role intervention, and triadic or family-centered interventions). We also provided nutritional counseling and neuropsychiatric monitoring of the overall process, including the effects of any pharmacological therapy. The aim of the present study was to look for significant pre-post differences in family functioning in the families of adolescent patients with severe REDs who underwent a 6-month (± two) multidisciplinary treatment program. Family functioning was assessed before and after the treatment using the LTPc procedure (13). Greater understanding of how family functioning may improve after a relatively brief multidisciplinary family treatment program may further inform effective interventions for these patients and their families. LTPc score changes are related to changes in family members' abilities to get involved in the game, to adhere to their assigned role in the different phases of it (and therefore, when necessary, to stand back), and to support others' ideas. Score changes may also be linked to greater emotional participation and exchange, as well as improved gaze triangulation.

## Materials and Methods

### Population

**Figure 1:**
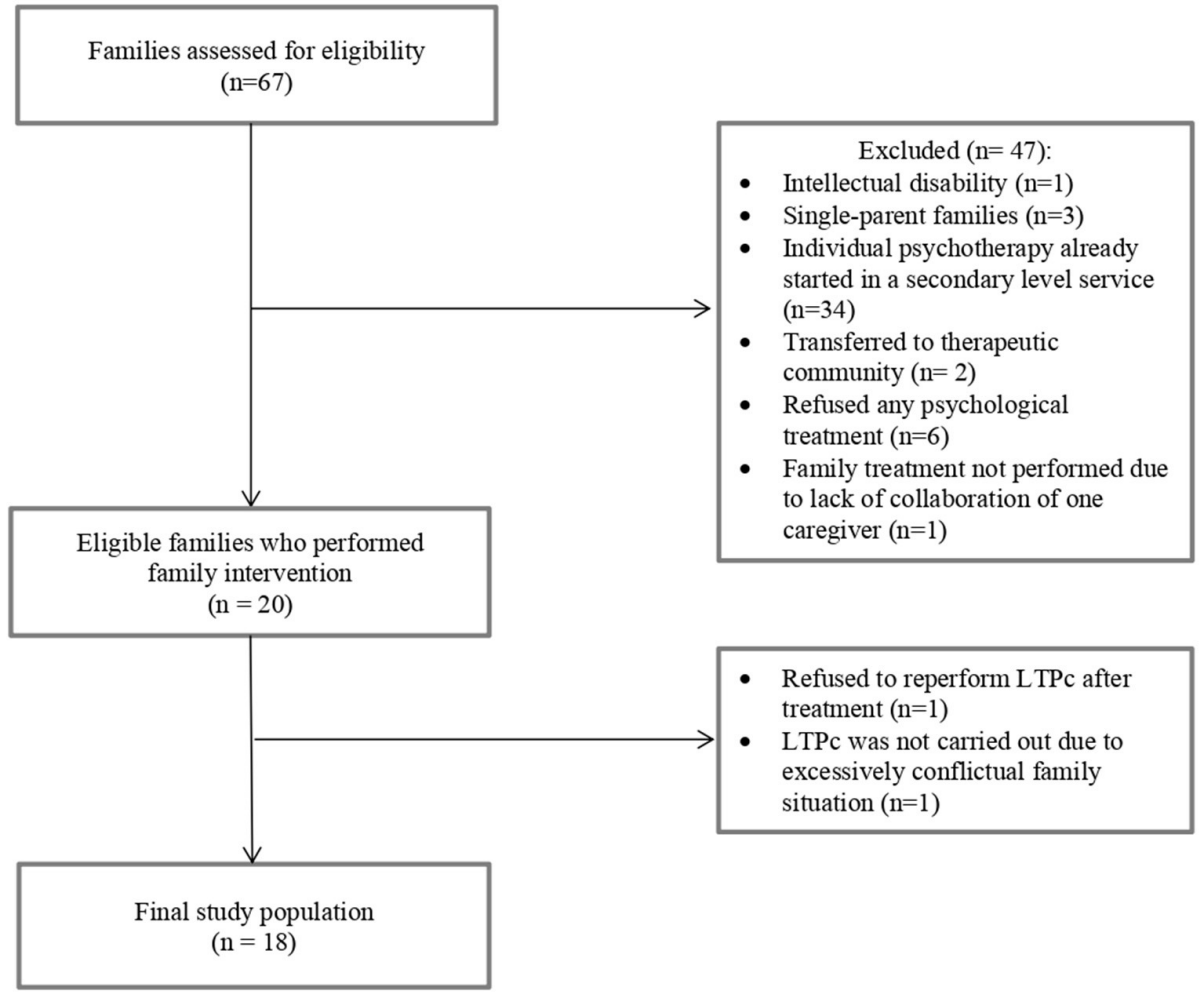
Flow chart of the study population.

Sixty-seven families of adolescent patients diagnosed with REDs were assessed for eligibility between July 2017 and October 2020 at the Child Neurology and Psychiatry Unit of the IRCCS Mondino Foundation (Pavia, Italy) and at the Child and Adolescent Neuropsychiatry Unit of the Bambino Gesù Children's Hospital (Rome, Italy). Patients were considered eligible for the study if they were 11–18 years old and if they had a diagnosis of RED (including restrictive and binge-eating/purging subtypes of anorexia nervosa, ARFID, atypical anorexia nervosa, other specified feeding or eating disorders with restrictive characteristics). Diagnoses were made according to the Diagnostic and Statistical Manual of Mental Disorders (DSM-5) criteria (42). Patients were excluded from the study if they presented at least one of the following: psychotic disorders, intellectual disability, neurological disorders (e.g., epilepsy), or other psychiatric comorbidities with an organic substrate (e.g., celiac disease, Wilson's disease). Single-parent families and individuals partially unable to understand Italian were also considered ineligible. Finally, to avoid interrupting or modifying ongoing therapies, we also excluded patients who were already receiving psychotherapy at a secondary-level service. The study was approved by the Ethics Committee of the Policlinico San Matteo in Pavia, Italy (P-20170016006). All the enrolled patients and their parents provided written informed consent to participate in the study. Figure 1 illustrates the flow chart of the participant selection process.

### Procedures

The patients were interviewed by a trained child neuropsychiatrist, who collected clinical and socio-demographic data. To confirm the RED diagnosis and verify the presence of any comorbidities, the semi-structured DSM-based K-SADS interview (43) was administered to the patients and their parents. Furthermore, the absence of intellectual disabilities was verified through administration of the age-appropriate Wechsler intelligence scale: WISC-IV (44) or WAIS-IV (45). To evaluate family functioning, the LTPc procedure (13) was used twice, before (T_0_) and after the treatment (T_1_). Every LTPc session, performed in a dedicated room, was videotaped and subsequently coded independently by two raters, who had first received specific training.

#### Treatment

**Figure 2:**
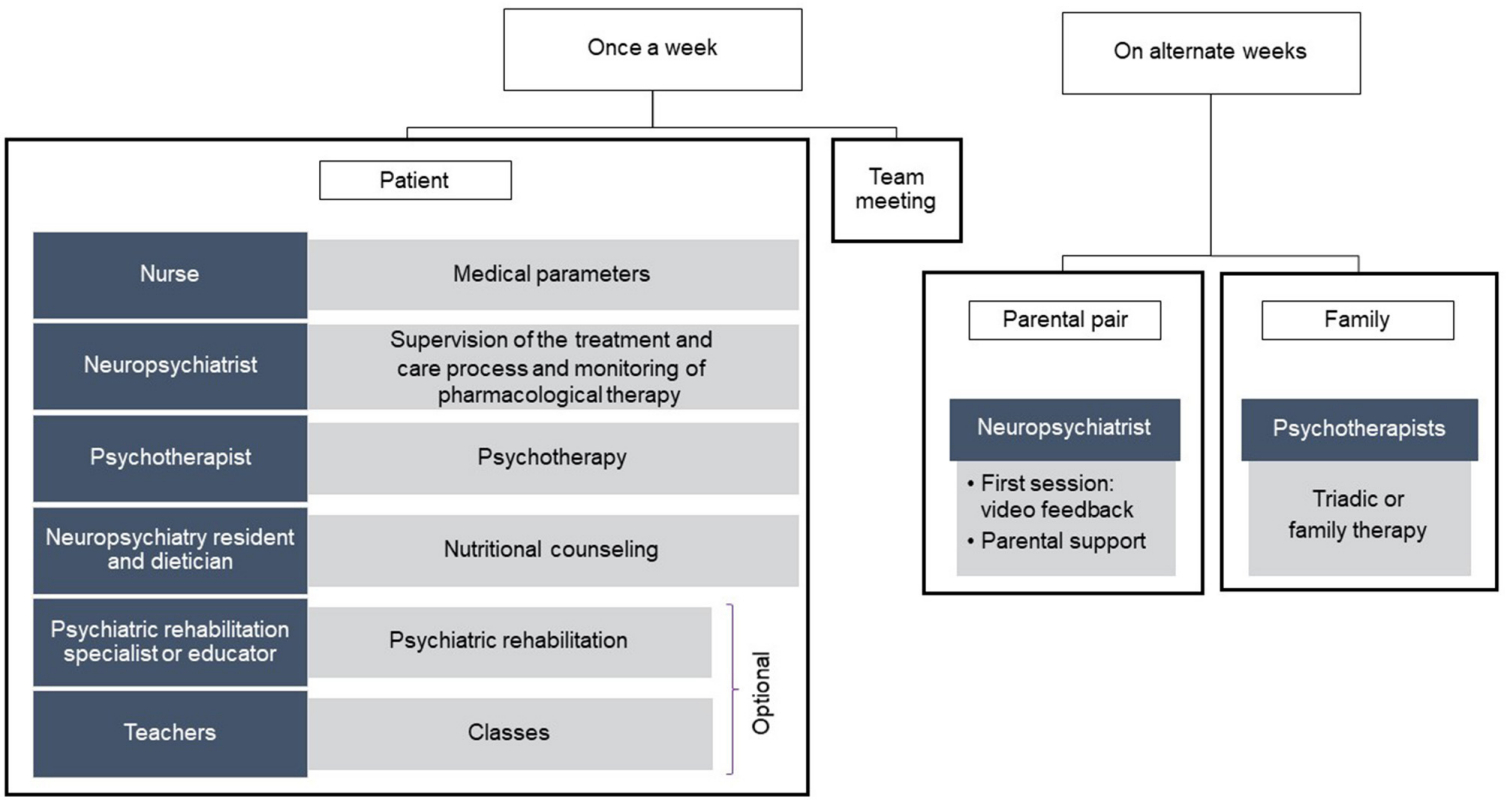
Overview of the multi-disciplinary intervention.

The treatment lasted 6 (±2) months and involved a multidisciplinary team (Figure 2), as the main international guidelines suggest that the care of patients affected by REDs should be entrusted to a team of medical, social, and rehabilitation healthcare professionals (24, 46).

Our multidisciplinary team comprised an expert neuropsychiatrist, a neuropsychiatry resident, psychotherapists, a psychiatric rehabilitation specialist, an educator, and a nurse.

The integrated treatment model (see Supplementary Material) consisted of at least 24 sessions of psychodynamic psychotherapy for the adolescent patient, scheduled once a week and conducted in an individual or group setting (40, 47, 48), at least 12 parental role intervention sessions (49, 50), and at least 12 treatment sessions focusing on triadic or family interaction. The parental role sessions took place every other week, alternating with the triadic or family interventions. The first session with the parents always involved the use of video feedback, which allows parents to work directly on their own limits and resources, favors the development of the ability to reflect on the relationship (mentalization), and significantly improves the therapeutic alliance (51–53).

Finally, nutritional counseling was provided, as well as neuropsychiatric monitoring of the progress of the treatment, to allow introduction or adjustment of pharmacological therapy as needed, as in the case of comorbid depressive or anxious symptoms. Further details on the intervention are reported in Supplementary Material.

#### The LTPc: Procedure and Coding

The LTPc is a standardized and well-validated observation-based method used in clinical and research settings to assess dysfunctional patterns in triadic or family interactions (13). The procedure requires parents and daughter to pretend that they are planning a weekend where the adolescent daughter stays home alone. The pretend play is divided into four phases. In phase 1, the mother interacts with the patient while the father assumes the role of observer. In phase 2, the father plans the activity with the patient while the mother observes. In phase 3, all the family members interact with each other. Finally, in phase 4, the parents talk together, while the adolescent assumes the role of observer. The entire process is videotaped and lasts ~15 min.

The LTPc coding system used in this study has been explained in previous publications (14, 52, 54, 55). Essentially, it considers four aspects of interaction (i.e., participation, organization, focal attention, affective contact), which are rated, in each phase, on a three-point Likert scale (0 = dysfunctional; 1 = partially functional; 2 = functional). On this basis, descriptions of each family member's interactive contribution and of the overall family functioning are obtained. The total family score, which identifies one of four types of family alliance, is the sum of the scores recorded by each family member in each phase (13).

### Statistical Analyses

The statistical analyses were conducted using IBM SPSS Version 21 for Windows. Descriptive statistics were calculated for each variable. To test for stability, we adopted the mean-level change method (56) and rank-order consistency method (57). To assess mean differences in LTPc scores, separate paired sample *t*-tests were computed for each LTPc phase (1, mother-daughter; 2, father-daughter; 3, triadic interaction; 4, parental pair).

## Results

Table 1 reports the descriptive statistics for this sample. Eighteen 11- to 17-year-old girls (*M* = 14.64 years, SD = 1.47) who were being cared for in day-hospital settings participated in the study with their parents. Eleven girls came from the Child Neurology and Psychiatry Unit of the IRCCS Mondino Foundation in Pavia (61.11%) and seven from the Child and Adolescent Neuropsychiatry Unit of the Bambino Gesù Children's Hospital in Rome (38.89%). Two of the 18 pairs of parents were divorced (11.10%). The average duration of symptoms prior to clinical referral was 13.32 months (SD = 11.33). The severity of the patients' clinical conditions was assessed using the MROAS and coded as: 0 = good outcome, 1 = intermediate outcome, and 2 = poor outcome. These outcomes were distributed as follows: 25.4% good, 44.1% intermediate, and 30.5% poor. At baseline (T_0_), the average percentage of weight loss reported by the patients was 22.02% (SD = 11.15), and their average BMI was 13.1 kg/m^2^ (SD = 18.74) (range: 11.91–32.11 kg/m^2^). The median pre-treatment percentile BMI was 1.2. Within the sample, 28 of 67 patients were using medications before T_0_ (i.e., 6.9% were taking antipsychotics, 58.6% antidepressants, 6.9% benzodiazepines, and 27.6% a combination of antipsychotics and antidepressants). The total family score in phase 2 (father–daughter) showed a statistically significant positive change from T_0_ to T_1_ (see Table 2). No significant differences emerged for the other LTPc phases.

**Table 1:** Descriptive statistics: patients' baseline diagnoses and therapies both before and during the multidisciplinary treatment program.

**Diagnosis**	***N***	**%**
**Eating disorders**		
Anorexia nervosa	13	72.22
Another restrictive eating disorder	5	27.78
**Schizophrenia**		
No	17	94.44
Yes^a^	1	5.56
**Depressive disorders^b^**		
No	8	44.44
Yes	10	55.56
**Anxiety disorders^c^**		
No	17	94.44
Yes	1	5.56
**Personality disorders**		
No	16	88.89
Yes	2	11.11
**Therapies in place before T**_**0**_	***N***	**%**
**Patient**		
No	2	11.11
Yes	16	88.89
**Parents**		
No	4	22.22
Yes	14	77.78
**Triadic or family therapy**		
No	4	22.22
Yes	14	77.78
**Interventions completed at T**_**1**_ **(all sessions completed)**	***N***	**%**
**Patient**		
No	0	0.00
Yes	18	100.00
**Parents**		
No	2	11.11
Yes	16	88.89
**Triadic or family therapy**		
No	4	22.22
Yes	14	77.78
**Dietary program**		
No	2	11.11
Yes	16	88.89

a *This diagnosis was made after enrollment in the study*.

b *Major Depressive Disorder, or Other Specified Depressive Disorder, or Unspecified Depressive Disorder*.

c *Separation Anxiety Disorder, or Other Specified Anxiety Disorder, or Unspecified Anxiety Disorder*.

**Table 2:** Descriptive statistics for the LTPc phases and mean comparisons.

	**T**_**0**_		**T**_**1**_		**Mean comparisons**		
**LTPc scores**	**Mean**	**SD**	**Mean**	**SD**	***t***	***p***	$\eta_{\text{p}}^{2}$
Phase 1 (mother-patient)	5.94	1.43	6.17	1.58	−0.58	0.57	0.019
Phase 2 (father-patient)	5.61	1.88	6.56	0.92	−2.36	0.03*	0.247
Phase 3 (mother-father-patient)	4.00	2.95	3.83	2.85	0.17	0.87	0.002
Phase 4 (mother-father)	5.22	2.05	4.78	2.65	0.77	0.45	0.034

*The family members involved in each LTPc phase are reported in brackets*.

Significance: *p < 0.05.

## Discussion

The aim of the present study was to assess post-treatment changes in family functioning among families of adolescents with severe REDs who underwent a multidisciplinary 6-month treatment program. We observed a significant change in the family functioning score for the LTPc phase 2, in which the father interacts with his daughter while the mother acts as a silent observer. This suggests that the fathers, when playing an active role, could improve dyadic family functioning. This finding is consistent with the idea, emerging from previous pioneering studies, that encouraging paternal involvement can improve patient outcomes (20, 21). In the families of girls affected by REDs, fathers tend to be disengaged from the caregiving role. Although this may be merely a defensive reaction to their daughter's illness, it can lead to a less affective bond and influence the quality of family interactions and the patient's outcome (15, 58). It can be speculated that the treatment model here proposed had more effect on the fathers than on the other members of the triad. In line with the current literature (59, 60), the results of our study therefore support the clinical indication of promoting affective engagement and participation of all family members, including fathers, in the care of adolescent patients, especially those with REDs (18, 19). A growing body of literature indeed suggests that therapeutic approaches to severe REDs in adolescence should include the promotion of paternal—and not only maternal—participation (20, 21, 61), in order to enhance the parents' alliance and improve the quality of triadic interactions. Paternal involvement and warmth have been shown to be fundamental for patient outcomes, and fathers who tend to draw back and remain emotionally and concretely detached need to be encouraged and supported (18, 20, 21, 62).

We did not find a similar change in maternal interactive behavior after the treatment. As others have pointed out (63–65), mothers are usually more involved in their daughters' afflictions. It is likely that a more prolonged family treatment would be needed in order to change dysfunctional interactive patterns in mothers. However, it can also be speculated that when fathers prove able to play a more active role, this may be due in part to mothers managing to give them more space (15).

We also found no post-treatment change in the functioning of the parental pair. This is in line with the fact that our treatment model, based on a psycho-pedagogical approach, was designed to strengthen the parental role rather than address relational dynamics (such as conflict and conflict management) between the parents themselves (66). Consequently, we were not surprised that the functioning of the parental pair remained unchanged after the treatment.

Finally, the treatment was not found to change triadic functioning. We can assume that a 6-month treatment is not long enough to modify interactions at the triadic level.

The lack of impact of the treatment on triadic functioning could also be explained by the fact that dyadic relations were highly impaired in our sample of adolescents; these were indeed patients whose psychopathological conditions were severe enough to warrant intervention by tertiary-level services.

This study has some limitations. First, the relatively small sample size (due to the need to include only triads with complete data and to the exclusion of patients already receiving psychotherapy) limits the generalizability of the findings. Future research in larger samples is needed. Second, we focused on REDs because the families of patients affected by these conditions frequently show dysfunctional family relations (15, 67). Future studies should investigate whether our results extend to other eating disorders. Finally, in line with the descriptive aim of this study, no control groups were included. Future research is warranted to address the relative effect of this multidisciplinary treatment program compared with care as usual and with other family- or patient-centered interventions.

## Conclusions

Since the psychopathological organization underlying REDs can vary, the therapeutic approach should be tailored to the specific features of the single patient. In particular, in the most severe cases, particular attention should be paid to parental (dyadic) and triadic or family interactions, but psychotherapy for patients only (individual or group) may also be very useful. We strongly suggest that a flexible therapeutic approach allowing integrated interventions (psychodynamic psychotherapy for patients, support for the parental role, and triadic or family intervention) might better meet the needs of the most impaired patients referred to tertiary care services. The LTPc may help clinicians to improve their understanding of dysfunctional family interactions and even uncover potential protective factors that might be further exploited to enhance the efficacy of the family intervention in RED patients (15). In addition, performing the LTPc after the treatment may assist the clinical decision-making process. For example, its findings may support the decision to continue with the current treatment or allow the treatment to be tailored to the needs of the family, perhaps suggesting a less-intensive program of treatment in order to obtain a better balance of family psychological and economic resources.

## Data Availability Statement

The raw data supporting the conclusions of this article will be made available upon reasonable requests to the corresponding author.

## Ethics Statement

The present study was reviewed and approved by Ethics Committee of the Policlinico San Matteo in Pavia, Italy (P-20170016006). Written informed consent to participate in this study was provided by the participants' legal guardian/next of kin.

## Author Contributions

MM designed the study. MO, CR, MCr, MCC, and VZ collected data. LP conducted statistical analyses. MCh and RB provided scientific supervision. All authors contributed to the drafting of the manuscript and agreed on the final version to be submitted for publication.

## Conflict of Interest

The authors declare that the research was conducted in the absence of any commercial or financial relationships that could be construed as a potential conflict of interest.

## Mondino Foundation Eating Disorders Clinical and Research Group

Luca Capone, Chiara Coci, Elisabetta Zerbi, Alice Busca, Maria Sabella, Lucia Racioppi.
