# Supplementary material for: Assessing Family Functioning Before and After an Integrated Multidisciplinary Family Treatment for Adolescents With Restrictive Eating Disorders
Source: Front Psychiatry. 2021 Jun 4;12:653047. doi: 10.3389/fpsyt.2021.653047 (PMC8211764; doi:10.3389/fpsyt.2021.653047)
Supplement: Supplementary file 1 [file Table_1.DOCX]

Integrated multidisciplinary family treatment in adolescents with restrictive eating disorders

As indicated by the main international guidelines (1,2), the care of people with restrictive eating disorders (REDs) must be entrusted to a multidisciplinary team composed of various specialists.

Accordingly, our team comprises different figures, to create a differentiating function within the family and the bonds between the adolescent and his/her parents in an active way and from the beginning of work.

Evaluating whether it is possible to constructively involve the family in the treatment is indeed a mandatory step in order to avoid failure of the treatment due primarily to an inappropriate or premature therapeutic choice (3,4).

Treatments often need to be tailored not only to the patient but also to each family’s needs (5–8).

Although it is the first choice in REDs, the systemic approach has not always proved effective, especially in the cases where it is not possible to constructively involve the family in the treatment (3,4).

On the other hand, if the family relationships are highly dysfunctional, individual psychotherapy can achieve only partial results.

Based on these premises, our psychiatric treatment and care model consists of:

- **Psychodynamic psychotherapy for adolescents**
- **Parental role intervention**
- **Triadic or family intervention**

| **Psychodynamic** **psychotherapy for adolescents** | |
| --- | --- |
| **Frequency** | Once a week |
| **Number of sessions** | At least 24 sessions |
| **Setting** | Individual or group |
| **Literature references** | - Adolescent-focused psychotherapy (9): a manualized version of ego‑oriented individual therapy. - Slavson (10) and Foulkes’ (11) group therapy models |
| **Team member** | One psychotherapist dedicated to patient or, in group setting, to patients’ group |
| **Objectives** | - self-exploration and development. - exploring all ways in which the concept of self plays a role in the maintenance of disease-specific factors. - learning to identify, define, and tolerate emotions to develop a more constructive coping style and improve self-efficacy. |

| **Parental role intervention** | |
| --- | --- |
| **Frequency** | Every other week, alternating with triadic therapy |
| **Number of sessions** | At least 12 sessions |
| **Setting** | Single parental pair or groups of parents |
| **Literature references** | Psycho-pedagogical intervention conducted with the parental pair, not including couple therapy, but working on parents’ emotionality and parental role (12–15). |
| **Team member** | One neuropsychiatrist dedicated to parental pair or, in group setting, to parents’ group |
| **Objectives** | - to support the maternal and paternal roles. - to learn how to express and understand one’s own and others’ emotional states. - to facilitate the process of separation and individuation, both by the adolescent but also by the parents. |

| **Triadic or family intervention** | |
| --- | --- |
| **Frequency** | Every other week, alternating with parental role intervention |
| **Number of sessions** | At least 12 sessions |
| **Setting** | Individual for every family |
| **Literature references** | Godart model (16) |
| **Team member** | Two psychotherapists with expertise in family therapy |
| **Objectives** | - promoting positive parenting. - promoting observational skills, especially those necessary to grasp the interpersonal relationship. - promoting empathy towards children. - building and maintaining the therapeutic alliance. - identifying areas of specific responsibility of the adolescent and defining the existing intergenerational boundaries between patient and family. - promoting the acquisition of skills that make it possible to protect and support the family unit. - allowing adequate expression and management of family conflicts. - allowing family to rediscover its own resources and strengths. - rebuilding family identity as a community. - allowing development of the patient’s autonomy. |

**References**

1. American Psychiatric Association. American Psychiatric Association Practice Guidelines for the treatment of psychiatric disorders: compendium 2006. American Psychiatric Publishing; 2006.

2. National Institute for Clinical Excellence (NICE). Eating Disorders: Recognition and Treatment. Full guideline. 2017;62:656–62.

3. Espie J, Eisler I. Focus on anorexia nervosa: modern psychological treatment and guidelines for the adolescent patient. *Adolesc Health Med Ther*. 2015;6:9. https://doi.org/10.2147/ahmt.s70300

4. Herpertz-Dahlmann B. Adolescent Eating Disorders: Update on Definitions, Symptomatology, Epidemiology, and Comorbidity. Vol. 24, Child and Adolescent Psychiatric Clinics of North America. W.B. Saunders; 2015. p. 177–96. https://doi.org/10.1016/j.chc.2014.08.003

5. Diamond-Raab L, Orrell-Valente JK. Art therapy, psychodrama, and verbal therapy: An integrative model of group therapy in the treatment of adolescents with anorexia nervosa and bulimia nervosa. Vol. 11, Child and Adolescent Psychiatric Clinics of North America. 2002. p. 343–64.

6. Gatta M, Lara DZ, Lara DC, Andrea S, Paolo TC, Giovanni C, et al. Analytical psychodrama with adolescents suffering from psycho-behavioral disorder: Short-term effects on psychiatric symptoms. *Arts Psychother*. 2010;37:240–7.

7. Gatta M, Gallo C, Vianello M. The Arts in Psychotherapy Art therapy groups for adolescents with personality disorders. *Arts Psychother*. 2014;41:1–6. https://doi.org/10.1016/j.aip.2013.11.001

8. Mannarini S, Boffo M, Balottin L. Beliefs about the patient’s role in the psychotherapeutic relationship: A latent trait perspective. *TPM - Testing, Psychom Methodol Appl Psychol*. 2013;20:277–94.

9. Fitzpatrick KK, Moye A, Hoste R, Lock J, Le Grange D. Adolescent focused psychotherapy for adolescents with anorexia nervosa. Vol. 40, Journal of Contemporary Psychotherapy. Springer; 2010. p. 31–9. https://doi.org/10.1007/s10879-009-9123-7

10. Slavson SR. An Introduction to Group Therapy. New York: The Commonwelth Fund; 1964.

11. Foulkes SH. Introduzione alla psicoterapia gruppoanalitica. Edizioni Universitaria Romane, editor. Roma; 1991.

12. Duclos J, Dorard G, Cook-Darzens S, Curt F, Faucher S, Berthoz S, et al. Predictive factors for outcome in adolescents with anorexia nervosa: To what extent does parental Expressed Emotion play a role? Jiménez-Murcia S, editor. *PLoS One*. 2018;13:e0196820. https://doi.org/10.1371/journal.pone.0196820

13. Hughes EK, Sawyer SM, Loeb KL, Le Grange D. Parent-Focused Treatment. In: Family Therapy for Adolescent Eating and Weight Disorders: New Applications. Routledge; 2015. p. 59–71.

14. Lafrance Robinson A, Dolhanty J, Stillar A, Henderson K, Mayman S. Emotion-Focused Family Therapy for Eating Disorders Across the Lifespan: A Pilot Study of a 2-Day Transdiagnostic Intervention for Parents. *Clin Psychol Psychother*. 2016;23:14–23. https://doi.org/10.1002/cpp.1933

15. Strahan EJ, Stillar A, Files N, Nash P, Scarborough J, Connors L, et al. Increasing parental self-efficacy with emotion-focused family therapy for eating disorders: a process model. *Pers Exp Psychother*. 2017;16:256–69. https://doi.org/10.1080/14779757.2017.1330703

16. Godart N, Berthoz S, Curt F, Perdereau F, Rein Z, Wallier J, et al. A randomized controlled trial of adjunctive family therapy and treatment as usual following inpatient treatment for anorexia nervosa adolescents. *PLoS One*. 2012;7. https://doi.org/10.1371/journal.pone.0028249
